# Supplementary material for: 18FDG-PET/CT-Scans and Biomarker Levels Predicting Clinical Outcome in Patients with Alveolar Echinococcosis—A Single-Center Cohort Study with 179 Patients
Source: Pathogens. 2023 Aug 14;12(8):1041. doi: 10.3390/pathogens12081041 (PMC10457873; doi:10.3390/pathogens12081041)
Supplement: Supplementary file 1 [file pathogens-12-01041-s001.zip › pathogens-2392043-supplementary.pdf]

Supplementary material

S1. Biomarkers in relation to clinical status

Patients without follow-up visit or with a follow-up period < 6 months were excluded.

Laboratory values	Cured	Stable without BMZ	Stable with BMZ	Progressive disease	Statistical analysis (Kruskal-Wallis-Test)
SAA, mg/L	n = 36	n = 37	n = 71	n = 6	$X^2(3) = 0.796$; $p = 0.850$
	M = 15.05<br>SD = 55.73<br>MD = 3.15<br>IQR = 4.28<br>Min = 0.80<br>Max = 335.00	M = 10.10<br>SD = 23.80<br>MD = 2.60<br>IQR = 10.60<br>Min = 0.80<br>Max = 146.00	M = 6.68<br>SD = 14.02<br>MD = 3.20<br>IQR = 4.60<br>Min = 0.80<br>Max = 115.00	M = 3.33<br>SD = 2.29<br>MD = 2.85<br>IQR = 3.83<br>Min = 0.80<br>Max = 7.10	
CRP, mg/L	n = 34	n = 36	n = 69	n = 6	$X^2(3) = 1.637$; $p = 0.651$
	M = 10.29<br>SD = 35.76<br>MD = 1.85<br>IQR = 5.93<br>Min = 0.20<br>Max = 210.30	M = 5.49<br>SD = 8.71<br>MD = 1.60<br>IQR = 5.95<br>Min = 0.30<br>Max = 44.00	M = 4.97<br>SD = 6.43<br>MD = 2.50<br>IQR = 4.70<br>Min = 0.20<br>Max = 29.30	M = 6.53<br>SD = 6.23<br>MD = 4.00<br>IQR = 12.25<br>Min = 0.60<br>Max = 15.40	
sIL-2R, U/ml	n = 36	n = 37	n = 71	n = 6	$X^2(3) = 0.621$; $p = 0.892$
	M = 320.00<br>SD = 183.40<br>MD = 261.50<br>IQR = 213.75<br>Min = 121.00<br>Max = 904.00	M = 291.43<br>SD = 168.18<br>MD = 243.00<br>IQR = 186.50<br>Min = 112.00<br>Max = 846.00	M = 310.90<br>SD = 194.35<br>MD = 266.00<br>IQR = 180.00<br>Min = 51.90<br>Max = 1252.00	M = 286.33<br>SD = 141.41<br>MD = 267.50<br>IQR = 169.25<br>Min = 147.00<br>Max = 554.00	
Ck18F(M30), U/L	n= 34	n = 36	n = 69	n = 6	$X^2(3) = 1.588$; $p = 0.662$
	M = 140.05<br>SD = 60.50<br>MD = 126.58<br>IQR = 42.25<br>Min = 30.57<br>Max = 349.04	M = 48.90<br>SD = 85.58<br>MD = 12.04<br>IQR = 90.14<br>Min = 68.91<br>Max = 523.85	M = 176.00<br>SD = 202.47<br>MD = 128.70<br>IQR = 82.88<br>Min = 66.92<br>Max = 1711.40	M = 151.53<br>SD = 39.46<br>MD = 159.07<br>IQR = 73.79<br>Min = 93.96<br>Max = 193.47	
Ck18F(M65), U/L	n = 34	n = 36	n = 69	n = 6	$X^2(3) = 2.960$; $p = 0.398$
	M = 275.20<br>SD = 269.28<br>MD = 184.06<br>IQR = 204.08<br>Min = 40.13<br>Max = 1142.70	M = 256.17<br>SD = 309.80<br>MD = 92.92<br>IQR = 200.27<br>Min = 37.49<br>Max = 1870.20	M = 344.29<br>SD = 543.83<br>MD = 200.00<br>IQR = 191.16<br>Min = 27.95<br>Max = 4199.60	M = 289.50<br>SD = 196.66<br>MD = 274.60<br>IQR = 334.50<br>Min = 70.99<br>Max = 605.12	
Ratio Ck 18F (M30:M65)	n = 34	n = 36	n = 69	n = 6	$X^2(3) = 1.512$; $p = 0.680$
	M = 0.91<br>SD = 0.75<br>MD = 0.63<br>IQR = 0.76<br>Min = 0.13	M = 0.99<br>SD = 1.08<br>MD = 0.72<br>IQR = 0.65<br>Min = 0.20	M = 0.82<br>SD = 0.75<br>MD = 0.63<br>IQR = 0.58<br>Min = 0.12	M = 0.74<br>SD = 0.43<br>MD = 0.64<br>IQR = 0.87<br>Min = 0.32	

	Max = 3.46	Max = 6.56	Max = 4.88	Max = 1.32	
ECP, µg/L	n = 34	n= 36	n = 69	n = 6	$X^2(3) = 0.281$; $p = 0.964$
	M = 4.37<br>SD = 3.46<br>MD = 2.85<br>IQR = 3.47<br>Min = 2.00<br>Max = 17.60	M = 3.85<br>SD = 3.05<br>MD = 2.95<br>IQR = 2.38<br>Min = 2.00<br>Max = 19.50	M = 3.90<br>SD = 2.71<br>MD = 3.10<br>IQR = 2.60<br>Min = 2.00<br>Max = 19.80	M = 4.25<br>SD = 2.52<br>MD = 2.95<br>IQR = 4.70<br>Min = 2.00<br>Max = 7.90	
EOS, G/L	n = 37	n = 38	n = 75	n = 7	$X^2(3) = 4.596$; $p = 0.204$
	M = 0.12<br>SD = 0.10<br>MD = 0.10<br>IQR = 0.10<br>Min = 0.00<br>Max = 0.30	M = 0.15<br>SD = 0.11<br>MD = 0.10<br>IQR = 0.10<br>Min = 0.00<br>Max = 0.50	M = 0.16<br>SD = 0.11<br>MD = 0.10<br>IQR = 0.10<br>Min = 0.50<br>Max = 2.20	M = 0.19<br>SD = 0.07<br>MD = 0.20<br>IQR = 0.10<br>Min = 0.10<br>Max = 0.30	
Specific IgE, kU/L	n = 37	n = 39	n = 75	n = 7	$X^2(3) = 38.564$; **$p < 0.001$**
	M = 1.16<br>SD = 3.58<br>MD = 0.12<br>IQR = 0.42<br>Min = 0.00<br>Max = 19.40	M = 1.50<br>SD = 5.47<br>MD = 0.14<br>IQR = 0.81<br>Min = 0.02<br>Max = 34.30	M = 4.08<br>SD = 7.00<br>MD = 1.57<br>IQR = 3.85<br>Min = 0.02<br>Max = 49.10	M = 5.38<br>SD = 7.94<br>MD = 0.69<br>IQR = 13.53<br>Min = 0.03<br>Max = 19.60	
Total IgE, IU/ml	n = 37	n = 39	n = 76	n = 7	$X^2(3) = 40.856$; **$p < 0.001$**
	M = 100.65<br>SD = 198.85<br>MD = 21.50<br>IQR = 97.85<br>Min = 3.40<br>Max = 987.40	M = 516.96<br>SD = 2313.18<br>MD = 45.60<br>IQR = 60.00<br>Min = 1.60<br>Max = 14089.00	M = 580.53<br>SD = 870.64<br>MD = 234.65<br>IQR = 526.57<br>Min = 1.10<br>Max = 5251.00	M = 1252.96<br>SD = 2106.86<br>MD = 103.00<br>IQR = 1897.90<br>Min = 3.80<br>Max = 5750.00	
IHA IgG, < 1:32 = '0'	n = 37	n = 38	n = 74	n = 7	$X^2(3) = 27.233$; **$p < 0.001$**
	MD = 0<br>P25 = 0<br>P75 =0<br>Min = 0<br>Max = 128	MD = 0<br>P25 = 0<br>P75 = 32<br>Min = 0<br>Max = 1024	MD = 32<br>P25 = 0<br>P75 = 128<br>Min = 0<br>Max = 4096	MD = 128<br>P25 = 0<br>P75 = 1024<br>Min = 0<br>Max = 1024	
Em2$^+$ negative	n = 33 (89.2%)	n = 23 (60.0%)	n = 20 (26.3%)	n = 1 (14.2 %)	Chi-Square-test: $X^2(3) = 44.805$; **$p < 0.001$**
Em2$^+$ positive	n = 4 (10.8%)	n = 14 (35.9%)	n = 55 (72.4%)	n = 6 (85.7%)	
PET negative*	/	n = 19 (48.7%)	n = 8 (10.5%)	n = 0 (0%)	Chi-Square-test: $X^2(2) = 23.925$; **$p < 0.001$**
PET positive*	/	n = 20 (51.3%)	n = 68 (89.5%)	n = 7 (100%)	

*Operated patients excluded

BMZ=Benzimidazoles; SAA=serum amyloid A; CRP=C-reactive protein; sIL-2R=soluble interleukin 2 receptor; Ck18F(M30) and Ck18F(M65)=cytokeratin fragments; EOS=total eosinophilic cell count; ECP=eosinophil cationic protein; specific IgE=parasite-specific IgE; total IgE=total immunoglobulin E; IHA=indirect hemagglutination IgG titers against a crude

parasite extract; $Em2^+$=antibody levels against Em2 antigen 2^+, if the index ≥1.0 will be considered to be positive, otherwise negative; PET positive=CT scans visually having FDG uptake around the AE lesions above the background liver uptake/non-affected liver tissue; PET negative=AE lesion with no detectable FDG uptake.

S2. Biomarkers and PET/CT results in relation to WHO stage of disease

Laboratory values	Stage I-II (P1/2N0M0)	Stage IIIa (P3N0M0)	Stage IIIb (P1-3N1M0 P4N0M0)	Stage IV (P4N1M0) P1-4N0/1M1)	Statistical analysis (Kruskal-Wallis-Test)
SAA, mg/L	n = 35	n = 45	n = 39	n = 49	$X^2(3)$ = 1.148; p = 0.765
	M = 9.17<br>SD = 20.10<br>MD = 2.50<br>IQR = 5.40<br>Min = 0.80<br>Max = 115.00	M = 13.41<br>SD = 49.44<br>MD = 2.80<br>IQR = 6.80<br>Min = 0.80<br>Max 335.00	M = 8.45<br>SD = 23.32<br>MD = 3.10<br>IQR = 2.50<br>Min = 0.80<br>Max = 146.00	M = 4.81<br>SD = 4.25<br>MD = 3.20<br>IQR = 4.90<br>Min = 0.80<br>Max = 18.70	
CRP, mg/L	n = 32	n = 43	n = 38	n = 49	$X^2(3)$ = 0.952; p = 0.813
	M = 4.60<br>SD = 6.23<br>MD = 1.65<br>IQR = 6.88<br>Min = 0.20<br>Max = 25.90	M = 10.10<br>SD = 32.48<br>MD = 2.10<br>IQR = 4.70<br>Min = 0.20<br>Max 210.30	M = 4.67<br>SD = 5.27<br>MD = 2.75<br>IQR = 6.00<br>Min = 0.20<br>Max = 22.40	M = 4.58<br>SD = 5.58<br>MD = 2.70<br>IQR = 4.35<br>Min = 0.30<br>Max = 29.30	
sIL-2R, U/ml	n = 35	n = 45	n = 39	n = 49	$X^2(3)$ = 2.897; p = 0.408
	M = 320.78<br>SD = 230.03<br>MD = 231.00<br>IQR = 173.00<br>Min = 132.00<br>Max = 1252.00	M = 293.66<br>SD = 155.11<br>MD = 278.00<br>IQR = 192.00<br>Min = 84.40<br>Max = 846.00	M = 266.63<br>SD = 150.11<br>MD = 229.00<br>IQR = 135.00<br>Min = 85.50<br>Max = 787.00	M = 378.70<br>SD = 343.91<br>MD = 286.0<br>IQR = 238.00<br>Min = 91.00<br>Max = 2181.00	
Ck18F(M30), U/L	n = 32	n = 43	n = 38	n = 49	$X^2(3)$ = 7.320; p = 0.062
	M = 133.28<br>SD = 63.73<br>MD = 111.65<br>IQR = 63.20<br>Min = 76.14<br>Max = 349.04	M = 138.17<br>SD = 61.80<br>MD = 128.69<br>IQR = 49.28<br>Min = 30.57<br>Max = 416.73	M = 152.45<br>SD = 80.97<br>MD = 132. 86<br>IQR = 64.94<br>Min = 67.80<br>Max = 488.52	M = 197.20<br>SD = 235.00<br>MD = 132.90<br>IQR = 97.90<br>Min = 73.86<br>Max = 1711.40	
Ck18F(M65), U/L	n = 32	n = 43	n = 38	n = 49	$X^2(3)$ = 4.496; p = 0.213
	M = 265.46<br>SD = 234.96<br>MD = 230.88<br>IQR = 227.95<br>Min = 51.10<br>Max = 1142.70	M = 214.03<br>SD = 155.95<br>MD = 176.04<br>IQR = 101.09<br>Min = 40.13<br>Max = 725.42	M = 267.87<br>SD = 326.08<br>MD = 169.24<br>IQR = 192.59<br>Min = 37.49<br>Max = 1988.40	M = 403.70<br>SD = 636.63<br>MD = 248.27<br>IQR = 226.87<br>Min = 37.49<br>Max = 4199.60	
Ratio Ck 18F (M30:M65)	n = 32	n = 43	n = 38	n= 49	$X^2(3)$ = 2.262; p = 0.520
	M = 0.78<br>SD = 0.57<br>MD = 0.54<br>IQR = 0.62<br>Min = 0.24<br>Max = 2.13	M = 0.95<br>SD = 0.76<br>MD = 0.73<br>IQR = 0.73<br>Min = 0.19<br>Max = 3.46	M = 0.95<br>SD = 0.73<br>MD = 0.83<br>IQR = 0.70<br>Min = 0.12<br>Max = 3.63	M = 0.87<br>SD = 0.98<br>MD = 0.53<br>IQR = 0.64<br>Min = 0.13<br>Max = 6.56	
ECP	n = 32	n = 43	n = 38	n = 49	$X^2(3)$ = 2.630;
	M = 3.66	M = 3.25	M = 4.34	M = 4.50	

	SD = 1.84<br>MD = 2.85<br>IQR = 3.15<br>Min = 2.0<br>Max = 7.70	SD = 1.39<br>MD = 2.00<br>IQR = 1.90<br>Min = 2.00<br>Max 7.70	SD = 3.29<br>MD = 3.10<br>IQR = 3.45<br>Min = 2.00<br>Max = 17.60	SD = 3.76<br>MD = 3.60<br>IQR = 3.00<br>Min = 2.00<br>Max = 19.80	$p = 0.452$
EOS, µg/L	n= 36	n = 47	n = 43	n= 50	$X^2(3) =$ 2.630; $p = 0.452$
	M = 0.14<br>SD = 0.11<br>MD = 0.10<br>IQR = 0.10<br>Min = 0.00<br>Max = 0.50	M = 0.16<br>SD = 0.14<br>MD = 0.10<br>IQR = 0.10<br>Min = 0.00<br>Max 0.90	M = 0.18<br>SD = 0.33<br>MD = 0.10<br>IQR = 0.10<br>Min = 0.00<br>Max = 2.20	M = 0.17<br>SD = 0.12<br>MD = 0.20<br>IQR = 0.13<br>Min = 0.00<br>Max = 0.50	
Specific IgE, kU/L	n = 37	n = 48	n = 42	n = 50	$X^2(3) =$ 10.442; **$p = 0.015$**
	M = 3.52<br>SD = 7.77<br>MD = 0.35<br>IQR = 1.43<br>Min = 0.00<br>Max = 34.40	M = 2.01<br>SD = 4.29<br>MD = 0.43<br>IQR = 1.78<br>Min = 0.02<br>Max = 21.10	M = 1.68<br>SD = 3.02<br>MD = 0.43<br>IQR = 1.72<br>Min = 0.01<br>Max = 12.50	M = 4.24<br>SD = 8.07<br>MD = 1.03<br>IQR = 3.93<br>Min = 0.03<br>Max = 49.10	
Total IgE, IU/ml	n = 37	n = 48	n = 42	Nn= 50	$X^2(3) =$ 14.137; **$p = 0.003$**
	M = 608.03<br>SD = 2311.86<br>MD = 57.00<br>IQR = 279.50<br>Min = 1.10<br>Max = 14089.00	M = 199.75<br>SD = 398.43<br>MD = 71.75<br>IQR = 97.48<br>Min = 2.20<br>Max 1941.00	M = 272.00<br>SD = 518.11<br>MD = 61.25<br>IQR = 260.67<br>Min = 1.14<br>Max = 2570.00	M = 755.64<br>SD = 1289.44<br>MD = 235.20<br>IQR = 572.82<br>Min = 2.20<br>Max 5750.00	
IHA IgG, < 1:32 = '0'	n = 35	n = 46	n = 41	n = 50	$X^2(3) =$ 13.705; **$p = 0.003$**
	MD = 0<br>P25 = 0<br>P75 = 32<br>Min = 0<br>Max = 1024	MD = 0<br>P25 = 0<br>P75 = 40<br>Min = 0<br>Max = 2048	MD = 0<br>P25 = 0<br>P75 = 32<br>Min = 0<br>Max = 8192	MD = 32<br>P25 = 0<br>P75 = 256<br>Min = 0<br>Max = 4096	
$Em2^+$ negative	23 (62.2%)	26 (54.2%)	20 (47.6%)	12 (24.0%)	Chi-Square: $X^2(3) =$ 16.472; **$p < 0.001$**
$Em2^+$ positive	13 (35.1%)	20 (41.7%)	21 (50.0%)	38 (76.0%)	
PET negative*	7 (35.0%)	14 (35.9%)	7 (19.4%)	4 (9.1%)	Chi-Square: $X^2(3) =$ 10.346; **$p < 0.016$**
PET positive*	13 (65.0%)	25 (64.1%)	29 (80.6%)	40 (90.9%)	

*Operated patients excluded

BMZ=Benzimidazoles; SAA=serum amyloid A; CRP=C-reactive protein; sIL-2R=soluble interleukin 2 receptor; Ck18F(M30) and Ck18F(M65)=cytokeratin fragments; EOS=total eosinophilic cell count; ECP=eosinophil cationic protein; specific IgE=parasite-specific IgE; total IgE=total immunoglobulin E; IHA=indirect hemagglutination IgG titers against a crude parasite extract; $Em2^+$=antibody levels against Em2 antigen 2^+, if the index ≥1.0 will be considered to be positive, otherwise negative; PET positive=CT scans visually having FDG

uptake around the AE lesions above the background liver uptake/non-affected liver tissue; PET negative=AE lesion with no detectable FDG uptake.

S3. Biomarkers in relation to PET/CT results

Patients who had undergone surgery (n = 38) were excluded from this analysis.

Laboratory values	PET/CT scan		Mann-Whitney-U-test
	positive	negative	
SAA, mg/L	n = 102	n = 31	U = 1357.50; *p* = 0.234
	M = 7.40<br>SD = 18.42<br>MD = 2.80<br>IQR = 4.72<br>Min = 0.80<br>Max = 146.20	M = 6.31<br>SD= 5.88<br>MD = 4.20<br>IQR = 6.90<br>Min = 0.80<br>Max = 25.50	
CRP, mg/L	n = 99	n = 30	U = 1361.00; *p* = 0.489
	M = 5.30<br>SD = 7.51<br>MD = 2.50<br>IQR = 4.90<br>Min = 0.20<br>Max = 44.00	M = 3.67<br>SD = 3.86<br>MD = 1.85<br>IQR = 5.18<br>Min = 0.20<br>Max = 14.30	
sIL-2R, U/ml	n = 102	n = 31	U = 1415; *p* = 0.489
	M= 328.83<br>SD= 277.60<br>MD= 264.00<br>IQR = 177.50<br>Min = 51.90<br>Max = 2181.00	M = 277.52<br>SD = 153.00<br>MD = 228.00<br>IQR = 178.00<br>Min = 112.00<br>Max = 774.00	
CK18F(M30), U/L	n =99	n = 30	U = 1400.00; *p* = 0.636
	M = 168.31<br>SD = 174.67<br>MD = 129.60<br>IQR = 78.70<br>Min = 66.92<br>Max = 1711.40	M = 147.35<br>SD = 72.83<br>MD = 126.12<br>IQR = 77.74<br>Min = 73.86<br>Max = 41673.00	
CK18F(M65), U/L	n = 99	n = 30	U = 1255.50; *p* = 0.201
	M = 325.77<br>SD = 494.44<br>MD = 198.41<br>IQR = 211.31<br>Min = 27.95<br>Max = 4199.60	M = 196.50<br>SD = 111.44<br>MD = 180.94<br>IQR = 133.79<br>Min = 37.49<br>Max = 540.90	
Ratio Ck 18F (M30:M65)	n = 99	n = 30	U = 1270.00; *p* = 0.231
	M = 0.89<br>SD = 0.78<br>MD = 0.63<br>IQR = 0.73<br>Min = 0.12<br>Max = 4.88	M = 1.05<br>SD = 1.14<br>MD = 0.77<br>IQR = 0.59<br>Min = 0.23<br>Max = 6.56	
ECP, µg/L	n = 99	n = 30	U = 1426.00; *p* = 0.741
	M = 3.89<br>SD = 2.85	M = 3.68<br>SD = 1.99	

	MD = 2.90<br>IQR = 2.70<br>Min = 2.00<br>Max = 19.80	MD = 3.00<br>IQR = 2.45<br>Min = 2.00<br>Max = 8.20	
EOS, G/l	n = 107	n = 32	U = 1469.00; $p = 0.200$
	M = 0.19<br>SD = 0.24<br>MD = 0.10<br>IQR = 0.10<br>Min = 0.00<br>Max = 2.20	M = 0.13<br>SD = 0.09<br>MD = 0.10<br>IQR = 0.10<br>Min = 0.00<br>Max = 0.30	
specific IgE, kU/L	N = 107	N = 33	U = 995.00; $\boldsymbol{p < 0.001}$
	M = 4.11<br>SD = 7.44<br>MD = 1.18<br>IQR = 3.37<br>Min = 0.01<br>Max = 49.10	M = 0.85<br>SD = 1.53<br>MD = 0.17<br>IQR = 0.82<br>Min = 0.02<br>Max = 7.63	
IgE total, IU/L	n = 107	n = 33	U = 785.50; $\boldsymbol{p < 0.001}$
	M = 706.05<br>SD = 1637.33<br>MD = 185.30<br>IQR = 548.90<br>Min = 1.14<br>Max 14089.0	M = 79.24<br>SD = 119.85<br>MD = 46.3<br>IQR = 72.85<br>Min = 1.10<br>Max 576.30	
IHA IgG,<br>< 1:32 = '0'	n = 105	n = 31	U = 1058.50; $\boldsymbol{p = 0.001}$
	MD = 32<br>P25 = 0<br>P75 = 256<br>Min = 0<br>Max = 8292	MD = 0<br>P25 = 0<br>P75 = 32<br>Min = 0<br>Max = 256	
$Em2^+$ negative	n = 27 (25.2%)	n = 21 (63.6%)	Chi-Square-test
$Em2^+$ positive	n = 78 (72.9%)	n = 10 (30.3%)	$X^2(1) = 18.511$; $\boldsymbol{p < 0.001}$

BMZ=Benzimidazoles; SAA=serum amyloid A; CRP=C-reactive protein; sIL-2R=soluble interleukin 2 receptor; Ck18F(M30) and Ck18F(M65)=cytokeratin fragments; EOS=total eosinophilic cell count; ECP=eosinophil cationic protein; specific IgE=parasite-specific IgE; total IgE=total immunoglobulin E; IHA=indirect hemagglutination IgG titers against a crude parasite extract; $Em2^+$=antibody levels against Em2 antigen 2^+, if the index ≥1.0 will be considered to be positive, otherwise negative;
